# Supplementary material for: Deep sequencing of Escherichia coli exposes colonisation diversity and impact of antibiotics in Punjab, Pakistan
Source: Nat Commun. 2024 Jun 18;15:5196. doi: 10.1038/s41467-024-49591-5 (PMC11189469; doi:10.1038/s41467-024-49591-5)
Supplement: Supplementary file 1 — Supplementary Information [file 41467_2024_49591_MOESM1_ESM.pdf]

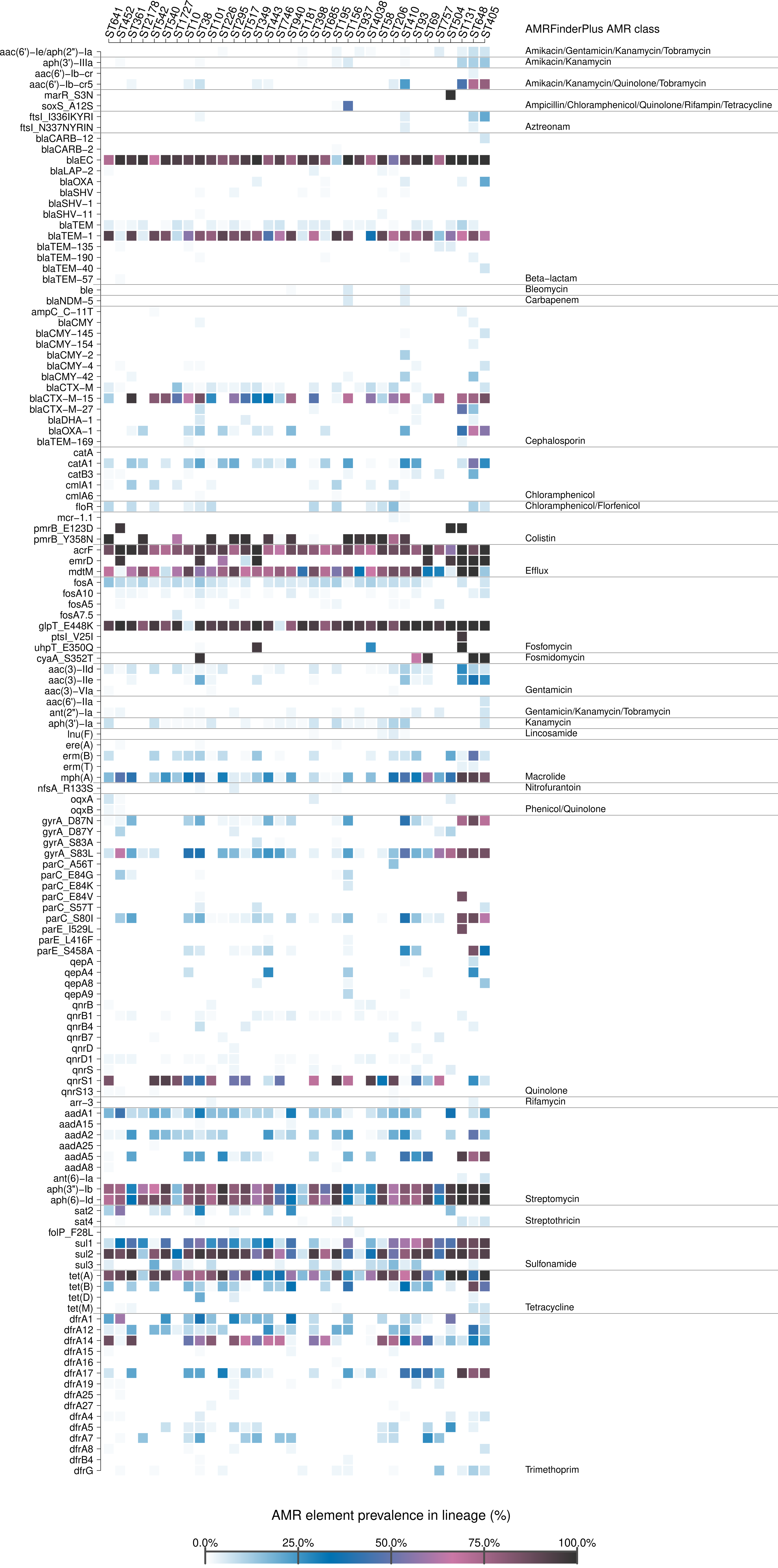

Supplementary Figure 1 All antimicrobial resistance determinants in the study Escherichia coli population. The plot shows the percentage of times an AMR determinant (rows) was found in a specific E. coli lineage (columns) out of the total number of observations of the lineage. Values are shown for the 30 most common lineages together with ST405, ST504, and ST543 which were all substantially enriched in the control samples. The plot shows only the AMR determinants that were observed at least five times in the included lineages and had a minimum sequence identity of at least 95% and coverage of at least 90% to a reference in the AMRfinder+ database using any method.
