# Supplementary material for: Deep sequencing of Escherichia coli exposes colonisation diversity and impact of antibiotics in Punjab, Pakistan
Source: Nat Commun. 2024 Jun 18;15:5196. doi: 10.1038/s41467-024-49591-5 (PMC11189469; doi:10.1038/s41467-024-49591-5)
Supplement: Supplementary file 3 — Description of Additional Supplementary Files [file 41467_2024_49591_MOESM3_ESM.pdf]

Description of Additional Supplementary Files

Supplementary Data 1: Contains information linking the patients and samples to the E. coli BAGs.

Supplementary Data 2: Contains quality control information for the E. coli BAGs plus a column "pass_QC" marking the 5,247 BAGs that were included in the presented analyses.

Supplementary Data 3: Contains information about what percentage of the individuals in each study cohort (Manga Mandi hospital primary samples, control samples, and the community samples) were colonised by a certain E. coli lineage. Colonisation by a lineage is defined as us successfully assembling a BAG from that lineage which passes the assembly quality control.

Supplementary Data 4: Lists antibiotic prescriptions each patient in the Manga Mandi hospital cohort received. Prescriptions were extracted from an electronic patient record system.

Supplementary Data 5: Links the full raw results from AMRFinderPlus (minimum identity 90% and coverage 95%) to the E. coli BAGs.
