# Supplementary material for: Deep sequencing of Escherichia coli exposes colonisation diversity and impact of antibiotics in Punjab, Pakistan
Source: Nat Commun. 2024 Jun 18;15:5196. doi: 10.1038/s41467-024-49591-5 (PMC11189469; doi:10.1038/s41467-024-49591-5)
Supplement: Supplementary file 9 — Reporting Summary [file 41467_2024_49591_MOESM9_ESM.pdf]

nature portfolio

Corresponding author(s): Jukka Corander

Last updated by author(s): May 27, 2024

Reporting Summary

Nature Portfolio wishes to improve the reproducibility of the work that we publish. This form provides structure for consistency and transparency in reporting. For further information on Nature Portfolio policies, see our Editorial Policies and the Editorial Policy Checklist.

Statistics

For all statistical analyses, confirm that the following items are present in the figure legend, table legend, main text, or Methods section.

n/a	Confirmed	
☐	☒	The exact sample size (n) for each experimental group/condition, given as a discrete number and unit of measurement
☐	☒	A statement on whether measurements were taken from distinct samples or whether the same sample was measured repeatedly
☐	☒	The statistical test(s) used AND whether they are one- or two-sided<br>*Only common tests should be described solely by name; describe more complex techniques in the Methods section.*
☐	☒	A description of all covariates tested
☐	☒	A description of any assumptions or corrections, such as tests of normality and adjustment for multiple comparisons
☐	☒	A full description of the statistical parameters including central tendency (e.g. means) or other basic estimates (e.g. regression coefficient) AND variation (e.g. standard deviation) or associated estimates of uncertainty (e.g. confidence intervals)
☐	☒	For null hypothesis testing, the test statistic (e.g. F, t, r) with confidence intervals, effect sizes, degrees of freedom and P value noted<br>*Give P values as exact values whenever suitable.*
☒	☐	For Bayesian analysis, information on the choice of priors and Markov chain Monte Carlo settings
☒	☐	For hierarchical and complex designs, identification of the appropriate level for tests and full reporting of outcomes
☒	☐	Estimates of effect sizes (e.g. Cohen's d, Pearson's r), indicating how they were calculated

Our web collection on statistics for biologists contains articles on many of the points above.

Software and code

Policy information about availability of computer code

Data collection	No software was used to collect the underlying sequencing data.
Data analysis	All software used in the study are freely available from sources indicated in the Methods section. Exact program calls and options used to run the analyses are available as Slurm batch job scripts from Zenodo (doi: https://doi.org/10.5281/zenodo.10077811). The scripts used for creating the figures in the manuscript is available at https://codeberg.org/themaklin/pakistan-e_coli-diversity-plots.<br><br>software used in the study fastp v0.23.2, themisto v3.0.0-rc, msweep v2.0.0, poppunk v2.5.0, coreutils demix check v0.3.2, mash v2.3, shovill v1.1.0, spades v3.15.5, checkm v1.2.1, assembly-stats c006b9c, amrfinderplus v3.10.42 with database 2022-10-11.2, gunc v1.0.5, ska2 v0.3.3, gubbins v3.2.1, and veryfasttree v3.3.0.

For manuscripts utilizing custom algorithms or software that are central to the research but not yet described in published literature, software must be made available to editors and reviewers. We strongly encourage code deposition in a community repository (e.g. GitHub). See the Nature Portfolio guidelines for submitting code & software for further information.

Data

Policy information about availability of data

All manuscripts must include a data availability statement. This statement should provide the following information, where applicable:

- Accession codes, unique identifiers, or web links for publicly available datasets
- A description of any restrictions on data availability
- For clinical datasets or third party data, please ensure that the statement adheres to our policy

The sequencing data used in the study is available from the ENA as part of project accession number PRJEB36642 and a list of the run accession numbers that originated from this study is included in Supplementary Table 1. The BAGs created in this study are available from Zenodo (doi: 10.5281/zenodo.10075671) and their associated metadata and assembly quality statistics are available in Supplementary Table 2 and Supplementary Table 3. The indexes used in the species and lineage level analyses are available from Zenodo (species index from doi: 10.5281/zenodo.6656881, E. coli lineage index from doi: 10.5281/zenodo.10077625).

Research involving human participants, their data, or biological material

Policy information about studies with human participants or human data. See also policy information about sex, gender (identity/presentation), and sexual orientation and race, ethnicity and racism.

Reporting on sex and gender	Participants were asked what their gender was. Although we were more interested in biological sex, the word "sex" was avoided by purpose in a conservative Muslim majority country. Three options were given: male, female and other. No participant answered "other".
Reporting on race, ethnicity, or other socially relevant groupings	Nothing about ethnicity was asked.
Population characteristics	Participants were asked their age. Median age was 38 years (IQR 28-50)
Recruitment	Outpatients were asked by their physicians to come visit the study site. In the communities we first contacted somebody known to us and who was respected in the community. For example in Yohanabad he was a Catholic priest, in Jalalpur Jattan just a well respected community dweller. The contact person then spread the word in the community that we were coming on a spesific date and asked those interested to approach us. After that the process was the same in the outpatient and community cohorts. Participants came to us, we orally explained what the study was about, gave participant information sheets and obtained written informed consent from those that were willing to participate and/or their guardians in case of minors. No compensation was paid. It is possible that those with abdominal symptoms were more likely to participate as we were taking stool/rectal swab samples.
Ethics oversight	Ethics committee of the School of Biological Sciences, University of the Punjab approved the study protocol (ref no: SBS/822/15)

Note that full information on the approval of the study protocol must also be provided in the manuscript.

Field-specific reporting

Please select the one below that is the best fit for your research. If you are not sure, read the appropriate sections before making your selection.

☒ Life sciences ☐ Behavioural & social sciences ☐ Ecological, evolutionary & environmental sciences

For a reference copy of the document with all sections, see nature.com/documents/nr-reporting-summary-flat.pdf

Life sciences study design

All studies must disclose on these points even when the disclosure is negative.

Sample size	We collected as many stool/rectal swab samples as was possible with the time and resources available. Sample size calculations were not relevant to our study setting.
Data exclusions	A total of 994 patients provided rectal swab samples at the Manga Mandi outpatient clinic. Of these, 497 delivered control samples, one was excluded for missing information on antibiotic use, and two for a possible mixup of samples. The final cohort thus included 494 patients
Replication	Sample collection and sequencing protocols are described under the Methods section of our manuscript. Scripts used to perform computational analysis of the data have been made publicly available.
Randomization	Participants were recruited from patients of all ages visiting the study hospital through referral by their physician, representing a random sample of the outpatient population. randomizing the participants to primary follow-up or community was not possible because of the nature of the study.

Blinding	Researchers were not blinded to group allocations due to the nature of the study which was reporting differences between the different groups.

Reporting for specific materials, systems and methods

We require information from authors about some types of materials, experimental systems and methods used in many studies. Here, indicate whether each material, system or method listed is relevant to your study. If you are not sure if a list item applies to your research, read the appropriate section before selecting a response.

Materials & experimental systems

n/a	Involved in the study
☒	☐ Antibodies
☒	☐ Eukaryotic cell lines
☒	☐ Palaeontology and archaeology
☒	☐ Animals and other organisms
☒	☐ Clinical data
☒	☐ Dual use research of concern
☒	☐ Plants

Methods

n/a	Involved in the study
☒	☐ ChIP-seq
☒	☐ Flow cytometry
☒	☐ MRI-based neuroimaging

Plants

Seed stocks	No seed stocks were used.
Novel plant genotypes	We do not report any plant genotypes.
Authentication	No seed stocks or plant genotypes are reported.
